# Spatial Heterogeneity in the Strength of Plant-Herbivore Interactions under Predation Risk: The Tale of Bison Foraging in Wolf Country

**DOI:** 10.1371/journal.pone.0073324

**Published:** 2013-09-11

**Authors:** Léa Harvey, Daniel Fortin

**Affiliations:** Département de biologie and Centre d'étude de la Forêt; Université Laval, Québec, Québec, Canada; Institut Pluridisciplinaire Hubert Curien, France

## Abstract

Spatial heterogeneity in the strength of trophic interactions is a fundamental property of food web spatial dynamics. The feeding effort of herbivores should reflect adaptive decisions that only become rewarding when foraging gains exceed 1) the metabolic costs, 2) the missed opportunity costs of not foraging elsewhere, and 3) the foraging costs of anti-predator behaviour. Two aspects of these costs remain largely unexplored: the link between the strength of plant-herbivore interactions and the spatial scale of food-quality assessment, and the predator-prey spatial game. We modeled the foraging effort of free-ranging plains bison (*Bison bison bison*) in winter, within a mosaic of discrete meadows. Spatial patterns of bison herbivory were largely driven by a search for high net energy gains and, to a lesser degree, by the spatial game with grey wolves (*Canis lupus*). Bison decreased local feeding effort with increasing metabolic and missed opportunity costs. Bison herbivory was most consistent with a broad-scale assessment of food patch quality, i.e., bison grazed more intensively in patches with a low missed opportunity cost relative to other patches available in the landscape. Bison and wolves had a higher probability of using the same meadows than expected randomly. This co-occurrence indicates wolves are ahead in the spatial game they play with bison. Wolves influenced bison foraging at fine scale, as bison tended to consume less biomass at each feeding station when in meadows where the risk of a wolf's arrival was relatively high. Also, bison left more high-quality vegetation in large than small meadows. This behavior does not maximize their energy intake rate, but is consistent with bison playing a shell game with wolves. Our assessment of bison foraging in a natural setting clarifies the complex nature of plant-herbivore interactions under predation risk, and reveals how spatial patterns in herbivory emerge from multi-scale landscape heterogeneity.

## Introduction

The interaction strength between a consumer and its resources is one of the most fundamental properties shaping food webs [1], [2]. Spatial variation in the strength of resource-consumer interactions can reflect adaptive decisions by foragers. Charnov's [3] landmark paper demonstrates how optimal foraging decisions can produce spatial structure in trophic interactions. The general principle is that no foraging opportunity is lost by remaining at a given feeding site until resource availability in that feeding site drops below a given threshold. Accordingly, foragers should experience fitness gains by consuming prey only at feeding sites where they experience at least the average net energy intake rate available in the landscape [3]. Brown [4] expanded the concept by predicting that local feeding effort should be adjusted to 1) the energy costs (C) of foraging, 2) the missed opportunity costs (MOC), such as those experienced by not foraging at a different site, and 3) the foraging costs of predation (P). Optimal foragers should leave a patch when their harvest rate (H) equals their foraging costs: H = C + MOC + P.

This framework has been successfully used to explain spatial variation in foraging effort by a number of species, such as Nubian ibex (*Capra nubiana*) [5], [6], white-tailed deer (*Odocoileus virginianus*) [7], mule deer (*O. hemionus*) [8], captive bison (*Bison bison*) and plains zebra (*Equus burchelli*) [9]. These studies generally evaluate the rate of forage gain in artificial food patches. The behaviour of free-ranging animals foraging on natural food in an unaltered setting, however, might provide more direct insight into the ecological determinants of spatial variation in the strength of trophic interactions. Following Brown's [4] framework, local foraging efforts in natural settings can be related to habitat covariates associated with each of the three foraging costs: C, MOC, and P.

### Energy costs of foraging (C)

Metabolic costs of foraging depend on a variety of habitat features influencing energy expenditures [4]. For example, environmental temperature influences the energy that homeotherms allocate to thermoregulation [10], [11]. Snow conditions can also impact foraging costs in temperate regions [12] by influencing the costs of searching, handling and extracting food from the snow. As a result, mule deer, plains bison (*B. b. bison*), elk (*Cervus canadensis*) and wombats (*Vombatus ursinus*) adjust their movements and local foraging efforts to snow depth and density [13]–[16], while muskoxen (*Ovibos moschatus*) change their pawing rate as snow accumulates [17].

### Missed opportunity costs (MOC)

Missed opportunity costs include any fitness enhancing activity given up while an individual forages in a particular feeding site, such as defending a territory, breeding, or foraging at another site [18]. Here we focus on foraging-related MOCs. When an individual lives in an environment rich in high-quality food, MOCs are high, and there should be fitness benefits to feed selectively and leave individual patches relatively soon [3], [19], [20]. For example, white-tailed deer and Angora goats (*Capri hircus*) respond to habitat enrichment by feeding more selectively on natural forage [19].

The foraging patterns of consumers result from behavioural adjustments to multi-level patterns of resource distribution [21], [22]. The food bites of a herbivore can be clustered into plant patches, which in turn, can be aggregated in foraging sites, and several of these sites ultimately comprise the animal's home range [23]. Accordingly, MOCs can be quantified at multiple levels [24]–[26]. At the landscape level (e.g., home range level for wide-ranging species), foraging-related MOC can be quantified as the difference between the average food profitability (profitability: E =  digestible energy/handling time) of all feeding sites available over the landscape and food profitability of the feeding site currently occupied (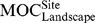 =  E_Landscape_ – E_Site_). Assuming that the number of feeding sites is large (i.e., negligible change of removing or adding one site on the average food profitability), average food profitability in the landscape can be seen as a constant [13], [25] related to the forager's long-term expectations. In this case, -E_Site_ can serve as a surrogate for 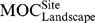 because the two variables would be directly proportional to one another.

Animals can also assess food quality based on their local foraging experience [13], [27]. MOC when foraging in a given plant patch within a feeding site can thus be quantified at two levels: 1) by comparing the patch to other patches available in the landscape (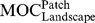 =  E_Landscape_ – E_Patch_), and 2) by comparing the patch to the vegetation available in the rest of the feeding site (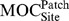 =  E_Site_ – E_Patch_). In the first case, 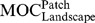 should be proportional to -E_Patch_, assuming that a large number of foraging patches are available in the landscape. This estimation of MOC would be consistent with McNamara et al.'s [28] contention that patch residency time (i.e., local foraging effort) depends on the long-term expectations of energy gain for rate-maximizing animals. In the second case, 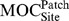 would reveal adjustments to the local potential of energy gain. This approach is more consistent with foragers making foraging decisions based on short-term sampling experiences [29]–[32]. Multi-level definition of MOCs has remained largely unexplored, but might be the key to understanding the spatial structure in foraging effort.

### Predation costs (P)

Foraging costs of predation (P) increase with predation risk, such as in habitats often used by predators [4], [33], [34] or where predators are most likely to succeed in capturing prey [35]–[38]. Prey are predicted to forage less in risky areas because they spend more time scanning their surroundings [8], [39]. The impact of predation risk on local feeding effort, however, may depend on the spatial game between predators and prey [40].

Prey have an evolutionary incentive to lessen the foraging costs of predation risk because of their fitness consequences [41], [42]. The prey's ability to do so may depend on their capacity to win the spatial game they play with predators. If prey can segregate themselves from predators, they are considered as being ahead in the spatial game, whereas a positive spatial association would imply that predators are the game's winners [40].

The predator-prey spatial game can take many forms. Prey may play a shell game by making frequent and relatively unpredictable movements across the landscape to prevent the predator from knowing their location [43]–[45]. When predators are unable to predict the location of their prey, they may benefit from surveying sites rich in their prey's food. This behaviour can drive prey out of these productive sites, and may result in a leapfrog effect, whereby predators select sites rich in their prey's food, while prey select poorer sites [40]. This situation more likely occurs when prey use a variety of habitats [46] and, therefore, do not have strong spatial constraints or anchor points within the landscapes. Leapfrog effects imply less intensive plant-herbivore interactions in productive patches comprised of highly profitable plant species. Prey would therefore not maximize their energy intake rate, and the relevant foraging currency would be more closely linked to a ratio between foraging gains and predation costs [47].

Alternatively, prey may simply undergo predation risk without being able (or willing) to segregate themselves from predators, resulting in the predator winning the spatial game [40]. This lack of response still allows the prey to play a shell game with their predators, in which case, both use patches rich in profitable vegetation for the prey, but not necessarily at the same time. Playing the shell game might lead to earlier departure than expected from energy-maximization principles, i.e., before patch depression [48]. The cumulative impact of herbivory on vegetation could then also strongly depend on the return rate of herbivores to individual patches. The nature of the spatial game between predator and prey can therefore largely control the strength of plant-herbivore interactions [46].

In this study, we evaluated the foraging dynamics of free-ranging plains bison during winter in Prince Albert National Park, Canada, by characterizing snow craters that bison dug to access the graminoids growing in meadows. In the park, high-quality forage for bison is organized in a multi-level mosaic: several plant communities differing in profitability (i.e., foraging patches) are found in meadows (i.e., feeding sites), which are imbedded in a forest matrix (i.e., landscape). We used a multivariate approach to model foraging efforts as a function of covariates associated with each of C, MOC and P. In the case of MOC, we tested the relative empirical support provided by different reference points used to assess food quality: global (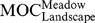 or 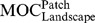) and local (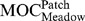) MOC. Finally, we also evaluated the wolf-bison spatial interaction by characterizing the spatial association between wolves and bison, using location data from GPS-collared individuals of both species.

## Methods

### Ethics Statement

The research was conducted in Prince Albert National Park, a Canadian National Park, in accordance with the research permit PA-2010–4552 provided by Parks Canada. The study was carried out in strict accordance with the recommendations in the Guide to the Care and Use of Experimental Animals of the Canadian Council on Animal Care. The protocol was approved by an Institutional Animal Care and Use Committee – Comité de protection des animaux of the Université Laval – Permit Number: 2008167–3. Bison collaring was performed under anesthesia (by injection of Carfentanil-Xylazine), whereas wolf collaring was done with individual under physical restraint. All efforts were made to minimize stress and suffering.

### Study area

The study was conducted in the southwestern corner of Prince Albert National Park (Saskatchewan, Canada). The area holds one of the few free-ranging populations of plains bison in North America. The bison occupy a landscape composed of forest (85%), meadows (10%), and water bodies (5%) [49]. The area included few roads that are accessible to park staff and researchers. The most abundant plants found in wet and mesic meadows consist of three tall plants (*Carex atherodes*, *C. aquatilis*, and *Scolochloa festucacea*), three mid-size plants (wheatgrasses, *Calamagrostis inexpansa* and *Juncus balticus*) and two short plants (*Hordeum jubatum* and *Poa* spp.). The main wheatgrasses are *Elymus trachycaulus* and *Pascopyrum smithii*. These nine plants constitute 95% of the bison diet in such meadows [49]. Multiple wolf packs are present in the study area. A number of bison show sign of altercation with wolves (e.g., missing tails), and since 2007, at least six of the radio-collared, adult female bison likely died from wolf predation (*Unpublished data*).

### Crater characteristics

During the winters of 1997, 1998 and 2011, we repeatedly surveyed 26 meadows (25 visited 4 times in 1997–1998 and 11 visited 1 to 3 times in 2011) for fresh foraging snow craters (i.e., those that were created since the previous visit) from 11 January to 27 March. We then estimated the area and the vegetation characteristics of each crater found. The area was estimated assuming simple geometric shapes [13]. In most cases, the crater was simply circular and its diameter was measured to estimate the area.

### Vegetation characteristics

#### Plant biomass

During the surveys, we estimated the biomass of vegetation available and the biomass consumed. Plant biomass available in each quadrat (0.25 m^2^) was visually scored on a 0–9 scale in 1997–1998 and on a 0–5 scale in 2011 (i.e., from small to large amount of vegetation). We established a relationship between plant biomass and visual scores after clipping above-ground vegetation in ≥30 random quadrats, then weighing the samples and allowing to dry for 60 h at 50°C [13]. The resulting relationships were: dry biomass (g/m^2^)  = 82.229e^0.29(visual estimate)^, R^2^ = 0.91, n = 39 for 1997–1998; and dry biomass (g/m^2^)  = 29.466e^0.5431(visual estimate)^, R^2^ = 0.81, n = 30 for 2011.

In 1998 and 2011, plant biomass consumed (g/m^2^) in a given crater was characterized based on 1 to 11 pairs of 0.25 m^2^ quadrats, depending on crater area (range: 1–5465 m^2^), and average values were used in subsequent analyses. A pair consisted of one quadrat inside the crater and one at 30 cm outside the crater where the plant community was similar (same species present in similar proportion) to the interior quadrat. Available biomass (*V*, g/m^2^) was estimated from the exterior quadrat, and the biomass remaining after the departure of bison was estimated from the interior quadrat. To obtain the plant biomass consumed, the remaining biomass was subtracted from the available biomass for each pair of quadrats.

#### Plant profitability

To assess the percent digestibility of the vegetation (%), we collected the top 40% of the vegetation layer [34], [50] in ungrazed quadrats. Samples were dried at 50°C for 60 h, ground, and their digestibility estimated following the methods of Tilley and Terry [51] with bovine rumen fluid [52]–[54]. Percent digestibility of vegetation was converted to digestible energy (*e*, kJ/g) using the following equation: *e* =  Digestibility (%) ×18.4096 kJ/g [53], whereas dry matter intake (F, g/min) was determined from our estimate of dry plant biomass (calculated from plant biomass estimated before grazing; V, g/m^2^) based on the functional response provided in Fortin et al. [54]:
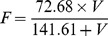



We then estimated the expected profitability (*E*, kJ/min) of the vegetation in a given quadrat as *e* (kJ/g) × *F* (g/min).

In 1997–1998, surveys were conducted by sampling 16–90 evenly spaced quadrats, adjusted to meadow area, covering the entire focal meadows [54]. The expected profitability of the vegetation was then calculated for the entire meadow as the average of the expected profitability of all quadrats in the meadow. In 2011, the sampling was done 1) for each snow crater (1–11 quadrats depending on crater area), and 2) randomly in the unused part of the meadow (10–15 quadrats depending on meadow area). Therefore, we estimated the expected profitability of the vegetation for the entire meadow (*E_Meadow_*, kJ/min) following a two-step procedure. First, the average profitability *E_Patch_* (kJ/min) for all *n* craters found in a given meadow followed:
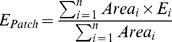
where *E_i_* is the profitability of the vegetation (kJ/min) in crater *i* among the *n* craters, and *Area_i_* is the area (m^2^) of crater *i*. The average profitability in the unused area *E_nc_* (kJ/min) was estimated as the average profitability calculated from the 10–15 quadrats randomly distributed in the meadow, outside foraging craters. Second, we estimated *E_Meadow_* as:
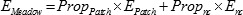
where *Prop_Patch_* is the proportion in the meadow area that is covered by craters, *Prop_nc_* is the proportion in the meadow area that is not covered by craters.

MOC were calculated differently, depending on the reference point considered to assess food quality. We used -*E_Meadow_* and *-E_Patch_* as surrogates for 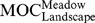 and 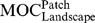, respectively, whereas we estimated 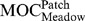 by subtracting the profitability expected within a patch to the profitability expected over the rest of the meadow (*E_Meadow_* – *E_Patch_*).

We estimated plant profitability in unused parts of each meadow after bison left the area. Following each vegetation survey, we determined the proportion of quadrats comprised of vegetation with a profitability (*E_quadrat_*) higher than the average profitability of all quadrats surveyed in the landscape (*E_Landscape_*).

### Snow characteristics

In 1997–1998, snow was characterized 1) at 1–4 locations (depending on crater area), at 30 cm of each crater in an area of undisturbed snow, and 2) in 16–90 evenly spaced quadrats, adjusted to meadow area, covering the entire focal meadow [54]. In 2011, snow depth and density was measured 1) at 1–5 locations (depending on crater area), at 30 cm of each crater in an area of undisturbed snow, and 2) at each of 10–15 vegetation quadrats, adjusted to meadow area and randomly distributed in the undisturbed snow areas of the focal meadows. Snow density (g/cm^3^, which also corresponds to the proportion of water per 1 cm^3^) was estimated by weighing, with a spring scale, a sample of the snow column collected using a metal tube, and dividing it by the volume of snow gathered. Snow depth was evaluated by measuring the height (in cm) of the snow column from the ground with a ruler. We then calculated snow water equivalent (SWE, cm) by multiplying the snow depth by its density [55]. SWE has the advantage of combining information on depth and density into a single estimate.

### Spatial association between wolves and bison

The relative use of the focal meadows by wolves was assessed during the crater surveys by recording the presence of wolf tracks. The presence of tracks in a given meadow was then considered a dichotomous variable taking a value of 1 when at least one track was observed in the meadow, and 0 otherwise.

To assess the spatial association between wolves and bison, we first estimated the distribution of radio-collared wolves over the bison range. We followed 8 wolves from 3 packs equipped with Global Positioning System collars (GPS collar 3300 from Lotek Engineering, Newmarket, ON, Canada) scheduled to take locations every 4 hours during the winters of 2008, 2009, 2011 and 2012. During the same four winters, we followed 23 female bison equipped with Global Positioning System collars (GPS collar 4400 from Lotek Engineering, Newmarket, ON, Canada) taking a location every 3 hours. The locations were used to assess the probability of wolf-bison co-occurrence.

### Data analysis

#### Probability of meadow use

We modeled the probability that bison use a particular meadow by relating meadow use (value of 1 if at least one foraging crater was present in the meadow, and 0 otherwise) to SWE, global missed opportunity costs (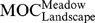), the index of wolf presence and meadow area using mixed effects logistic regression (GLIMMIX, SAS 9.2, SAS Institute Inc. 2008), with an adaptive Gaussian quadrature procedure to obtain accurate log-likelihood approximations [56]. Meadow and year were considered as random effects, and model performance was assessed based on the area under the Receiver Operating Characteristic curve (AUC) [57].

#### Probability of wolf-bison co-occurrence

To assess the spatial association between wolves and bison, we first estimated the wolf utilization distribution (UD) using bivariate normal kernels, with smoothing parameter h_ref_ and a 10 m resolution [58]. Because GPS locations from individuals of the same pack were non-independent, we calculated a UD for each wolf, and then averaged those values among pack members to obtain a single UD per pack. Each pack-level UD was then standardized between 0 and 100 to quantify the relative use of the landscape by wolves. When packs were overlapping, pack-level UDs were summed to account the additive effect of two wolf packs in the same area.

We tested if the probability of occurrence of bison varied with wolf UD. To do so, 1) we determined the wolf UD value for every bison location within the area occupied by radio-collared wolves (n = 9 358), 2) we randomly drew 9 358 random locations within the same area and 3) we determined the UD value for those random locations. We then related the observed and random locations using mixed effects logistic regression (GLIMMIX, SAS 9.2, SAS Institute Inc. 2008), with an adaptive Gaussian quadrature procedure [56]. Year and individual bison were considered as random effects and the robustness of the model was based on *k*-fold cross-validation [59]. With this approach, higher Spearman's rank correlation coefficients (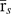) are indicative of more robust models; details are provided in [59].

#### Foraging intensity

To explain the spatial variation in foraging intensity, we tested the relationship between total area of snow craters in a given meadow and SWE, 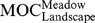, the index of wolf presence, and meadow area. We also estimated the relationship between the proportion of the meadow comprised of craters (arcsine[total area of snow craters/meadow area]^0.5^) and SWE, 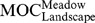, the index of wolf presence, and meadow area.

Models of total area of snow craters in meadows and the proportion of the meadow comprised of craters were both estimated using a linear mixed-effects model (MIXED, SAS 9.2, SAS Institute Inc. 2008; Gaussian distribution), with meadow and year as random effects. Model fit was assessed based on the pseudo R^2^ statistic defined as the square of the Pearson correlation statistic between marginal predictions and observed values [60].

#### Plant biomass consumed in a crater

Finally, we tested the relationship between the average plant biomass consumed in individual craters and SWE, 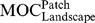 or 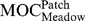, the index of wolf presence, and meadow area, using a linear mixed-effects model (MIXED, SAS 9.2, SAS Institute Inc. 2008; Gaussian distribution), with meadow and year as random effects. We evaluated the level of empirical support by the two models (i.e., one with 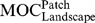 and the other with 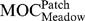) based on AIC [61]. Model fit was assessed based on the pseudo R^2^ [60].

#### Unused forage profitability

The relationship between the proportion of quadrats surveyed in unused parts of meadows with negative MOC (E_Landscape_ < E_quadrat_) and meadow area was tested using logistic regression (GLIMMIX, SAS 9.2, SAS Institute Inc. 2008) with an adaptive Gaussian quadrature procedure [56]. Meadow was considered as a random effect. When year was also included as a random factor, the model did not converge. We thus included year as a fixed effect.

All covariates of every statistical model had a variance inflation factor (VIF) <2, which indicates that multicollinearity was weak and statistical inference valid [62]. For all models, we also log-transformed the data whenever needed to linearize the relationship. Data are deposited in the Dryad repository: http://dx.doi.org/10.5061/dryad.4dp00.

## Results

### Probability of meadow use

Our model explaining whether or not bison create feeding snow craters in meadows had “good” predictability, with an AUC  = 0.84. The model indicated that the probability of bison foraging in a meadow (i.e., created at least one feeding crater) increased with (log-transformed) meadow area (Table 1). Further, meadows in which bison foraged were more likely to be visited by wolves. We did not detect any significant relationship with MOC or SWE (Table 1).

**Table 1:** Coefficients and standard errors for a mixed-effects logistic regression model predicting the probability that bison foraged in a given meadow in winter.

Variable (unit)	β	SE	P
Intercept	−1.41	1.67	0.41
Snow water equivalent (cm)	0.06	0.09	0.48
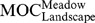 (kJ/min)	−0.003	0.004	0.51
Wolf presence	1.91	0.96	0.05
Ln(Meadow area, ha)	0.46	0.19	0.02
**Random effects**	**Variance**	**SE**	
Intercept	0.49	0.47	
Year	0.32	0.55	

Independent variables included snow water equivalent, missed opportunity costs of foraging in that meadow and not elsewhere in the landscape (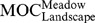), index of wolf presence (absence  = 0, presence  = 1) and log-transformed meadow area. N = 221 surveys in 26 meadows in Prince Albert National Park (Saskatchewan, Canada) during the winters of 1997, 1998 and 2011.

### Foraging intensity

Once foraging in a meadow, bison created larger craters if the 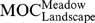 of foraging in that meadow was relatively low (Table 2). Feeding intensity further increased in meadows with relatively low SWE. Bison also created larger crater areas in larger meadows (Table 2), but this increase was at a diminishing rate. First, the increase relationship was stronger (ΔAIC  = 14.1) with log-transformed meadow area (AIC  = 465.2) than without any transformation (AIC  = 470.3). Second, the proportion of meadows comprised of craters (Prop) decreased with meadow area: arcsine(Prop)^0.5^ = 0.25−0.02(SWE) −0.0007(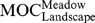) +0.04(Wolf Presence) −0.08ln(Meadow Size) (n = 144 and Pseudo R^2^ = 0.19, all coefficients had P<0.05, except for wolf presence and intercept). These findings indicate that, although bison make larger craters in large than small meadows, there was a larger area without foraging activity in large meadows. Moreover, the proportion of ungrazed feeding stations with profitability higher than the landscape's average was similar in small and large meadows (covariable ln[Meadow Size]: F_1,29_ = 0.41, P = 0.53), regardless of the year (interaction ln[Meadow Size] × year: F_2,29_ = 0.37, P = 0.70).

**Table 2:** Coefficients and standard errors of a linear mixed effects model predicting the area (ha) of foraging crater in individual meadows in winter.

Variable (unit)	β	SE	P
Intercept	−1.25	0.80	0.14
Snow water equivalent (cm)	−0.13	0.05	0.008
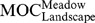 (kJ/min)	−0.005	0.001	0.003
Wolf presence	0.95	0.29	0.001
Ln(Meadow area, ha)	0.40	0.11	<0.001
**Random effects**	**Variance**	**SE**	
Intercept	0.20	0.13	
Year	0.01	0.12	

Independent variables included snow water equivalent, missed opportunity costs of foraging in that meadow and not elsewhere in the landscape (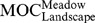), index of wolf presence (absence  = 0, presence  = 1) and log-transformed meadow area. A total of 144 foraging craters were recorded in 26 meadows in Prince Albert National Park (Saskatchewan, Canada) during the winters of 1997, 1998 and 2011. Pseudo R^2^ = 0.31.

Finally, we found that bison foraged more intensively in meadows where we found wolf sign than where we did not (Table 2). This relationship indicates that both species made intensive use of the same meadows. Indeed, using radio-telemetry, we found that the relative probability of occurrence of radio-collared bison (p) increased with the utilization distribution of radio-collared wolves (UD): p/(1−p)  = −0.43+2.57(UD) (P<0.0001; n = 9 358 bison locations for 23 female bison). This model was robust to cross-validation (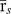 = 0.82±0.10).

### Plant biomass consumed in individual craters

The model of plant biomass consumed in individual craters with the most empirical support included missed opportunity costs at a global rather than a local scale (Table 3). The top-ranking model (H1, Table 3) indicates that bison tended to eat less vegetation, hence showed relatively low feeding efforts, in patches where global missed opportunity costs (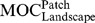) and SWE were high, and to a lesser degree, where there was evidence of wolf presence (Table 4).

**Table 3:** Relative level of support by competing models explaining plant biomass consumed in foraging craters by plains bison in winter.

Hypothesis	Model	AIC	ΔAIC
H1: E_Landscape_ - E_Patch_	SWE + 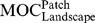 + Wolf + ln(MS)	3020.1	0
H2: E_Meadow_ - E_Patch_	SWE + 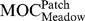 + Wolf + ln(MS)	3026.9	6.8

**Note:** E: Plant profitability (kJ/min) in the landscape, meadow or patch, SWE: Snow water equivalent (cm), MOC: Missed opportunity costs (kJ/min), Wolf: Index of wolf presence, ln(MS): log-transformed meadow size (ha), ΔAIC: difference in Akaike information criterion between the current model and the lowest AIC.

**Table 4:** Coefficients and standard errors for the top-ranking mixed effects linear model predicting the plant biomass consumed (g/m^2^) in a foraging crater in winter.

Variable (unit)	β	SE	P
Intercept	42.21	29.57	0.18
Snow water equivalent (cm)	−11.62	2.80	<0.0001
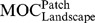 (kJ/min)	−0.56	0.05	<0.0001
Wolf presence	−33.16	18.39	0.07
Ln(Meadow area, ha)	−13.98	7.28	0.06
**Random effects**	**Variance**	**SE**	
Intercept	858.27	701.84	
Year	0	0	

Independent variables included snow water equivalent, missed opportunity costs of foraging in that meadow and not elsewhere in the landscape (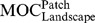), index of wolf presence (absence  = 0, presence  = 1) and log-transformed meadow area. A total of 255 quadrats of plant biomass were assessed in individual craters comprised in 23 meadows in Prince Albert National Park (Saskatchewan, Canada) during the winters of 1998 and 2011. Pseudo R^2^ = 0.66.

## Discussion

By relating feeding efforts to energy (C), missed opportunity (MOC) and predation (P) costs of foraging, we showed that the strength of plant-bison interactions is largely driven by the search of bison for high net energy gains (C and MOC) and, to a lesser extent, by their management of predation risk. Our assessment of the foraging behaviour of free-ranging animals in a natural setting underscores the complex nature of the trophic interactions, and reveals spatial determinants of plant-herbivore interaction strengths.

### Energy costs of foraging (C) and meadow attributes

Snow water equivalent (SWE) did not influence the probability that bison used a particular meadow. Once in a meadow, however, bison foraged over smaller areas if SWE was relatively high, and consumed less vegetation in craters where SWE was relatively high, a result consistent with previous reports [13], [49], [63]. A high SWE imposes a high energy cost [12], [64], [65], and large herbivores tend to adjust foraging efforts to spatial patterns in snow conditions [13], [15], [17].

To reduce travel costs and increase the rate of energy intake, bison should benefit from foraging more intensively in areas where highly profitable food is concentrated. This objective can explain the influence of landscape physiognomy on plant-herbivore interactions. Bison were more likely to use large than small meadows, and they used larger meadows more intensively. Factors controlling meadow characteristics thus determine, to a certain extent, the strength of bison herbivory. The shape and size of meadows in Prince Albert National Park result from ecological succession that followed the retreat of Laurentide glacier during late Pleistocene [66], [67]. Meadow dynamics are now linked to multiple processes. Beaver activity can create or maintain meadows [68]–[70], and fire helps maintain meadows by eliminating woody plants [71]–[73]. In absence of a natural fire and beaver activities, meadows gradually decrease in size due to tree and shrub encroachment [71], [72], a process that induces a gradual loss of high-quality foraging patches for bison. Spatial variation in the strength of bison-plant interactions are thus linked to abiotic (e.g., fire and geological processes) and biotic factors (e.g. shrub encroachment, beaver activity), through their influence on meadow dynamics.

### Missed opportunity costs (MOC)

MOC did not influence whether or not bison used a particular meadow. Once in a meadow, however, they fed more intensively (i.e., larger area covered by craters) if 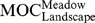 were relatively low. The average profitability of vegetation patches differed strongly among meadows. In 1998, for example, profitability averaged 401±123 kJ/min (mean ± SD, n = 23), with values ranging from 80 to 542 kJ/min in individual meadows. With such 6-fold differences among meadows, patch profitability can impact foragers in many ways. For example, bison should have to spend more time searching for profitable patches in poor versus rich meadows, experience stronger competitive interactions for the few rich patches available in poor meadows, and spend less time in poor meadows. Consistently, bison remain longer each time they enter meadows offering more of the highly profitable slough sedge (*C. atherodes*) [49], [74] and spend more total time in these meadows over a season [52].

We contrasted two reference points that foragers can use to assess local food quality: global MOC (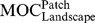) and local MOC (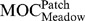). We found that the accumulation of craters in a meadow was best explained by broad-scale MOC (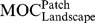), which supports the general idea that residency time at feeding sites should depend on the broad-scale expectations of long-term energy gains [28]. This observation does not appear consistent, however, with foragers adjusting their decisions based on short-term sampling experience [13], [29]–[31]. Bison are among those foragers, where they adjust their feeding intensity based on the vegetation encountered over the past 2 m^2^ of digging in the snow [13]. Therefore, these short-term foraging decisions should relate to local MOC (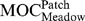). Considering this information and our current observations, we suggest that bison assess their MOC based on the information they acquire along their foraging path [13], but over time, the accumulation of foraging activities results in the most profitable foraging sites of the landscape being used more intensively (i.e., bison herbivory most consistent with 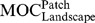). In other words, short-term processes could accumulate over time and result in long-term resource-consumer interactions that seemingly reflect other currencies. In any case, we show that MOCs decrease the intensity of herbivory, as the theory predicts [4].

### Predation costs (P)

Bison had a higher probability of occurrence in areas of high, rather than low, UD of wolves, and both species made strong use of meadows with low MOC for bison. The positive spatial association found between wolves and bison at broad-scales indicates that bison might not be able to, or willing to, segregate themselves from wolves, allowing the wolves to win the spatial game [40]. The observation that wolves were more likely to visit meadows rich in highly profitable bison forage could reflect an adaptive response to an elusive prey. Selecting patches of its prey's resource may be less costly than trying to track a highly mobile prey [43], [75], [76].

Contrary to elk in Yellowstone National Park (USA) during summer [77] and winter [78], and boreal caribou (*Rangifer tarandus caribou*) in Québec (Canada) during autumn and winter [79], bison did not select land cover types that would allow them to avoid wolves. Undermatching resource distribution to avoid predators may not always outweigh the fitness costs of reducing the use of food-rich patches [40]. Such a situation may occur when alternative food patches are of poor profitability. In Prince Albert National Park, graminoid biomass is more than 7 times higher in meadows than in forests [80]. Meadows would thus be a strong spatial anchor [40], which generally increases the likelihood of spatial associations between prey and predators [81].

Prey may still use multiple anti-predator tactics. For example, bison tended to consume less biomass at individual feeding stations in meadows where the arrival of a wolf was more likely (i.e., where we observed their tracks). Accordingly, behavioural observations in winter indicated that bison decrease their selection for highly profitable plant species in meadows that wolves use heavily [63]. This pattern may reflect foraging under the apprehension of an attack, where bison would then reduce their attention devoted to feeding and reallocate their attention to detecting wolf presence. The long-term impact of bison herbivory in presence versus in absence of predators would then depend on the accumulation of foraging activities over time at any given location.

Bison appeared to play a shell game with wolves by being constantly on the move. Although bison dug larger craters in larger meadows, there was a larger area without foraging activity in large than in small meadows. This result does not support optimal foraging theory based on the maximization of energy intake rate [3], [20], [54], which predicts an animal should feed at a site as long as its rate of energy intake does not drop under a given threshold. Bison left larger meadows when there were more areas of high-quality vegetation with no previous foraging activities than in small meadows. This observation is consistent with previous reports that bison consume only a small proportion of *C. atherodes* before leaving an area [74]. The proportion of meadows comprised of craters should not necessarily vary with meadow area, but with the energy gain function, which is likely to increase rather linearly over time for large mammalian herbivores [82], [83]. The fact that larger meadows were characterized by a lower proportion of craters than small meadows likely indicates that bison stopped foraging before their instantaneous intake rate of digestible energy dropped to the long-term expected rate. This early departure from meadows has already been suggested by Fortin et al. [84] and Courant and Fortin [74], and would be consistent with the predictions of a shell game [43], [44].

## Conclusion

Our study demonstrates that the search for high rates of net energy intake by bison results in spatial heterogeneity in the intensity of plant-herbivore interactions. Foraging decisions by bison depended on structural features of the landscape, with individuals increasing foraging intensity with meadow size, but at a decelerating rate. The relatively early departure from large meadows is consistent with the notion that bison do not spend too much time at any one place because they are involved in a shell game with wolves. This anti-predator tactic gives bison the opportunity to use highly profitable patches even if wolves also frequent the same patches. Given the absence of broad-scale segregation between wolves and bison, however, predation risk appears unlikely to have cascading effects on plant community through trait-mediated indirect interactions [46], [85].
